# *Propionibacterium acnes* Induces Intervertebral Disc Degeneration by Promoting iNOS/NO and COX-2/PGE_2_ Activation via the ROS-Dependent NF-*κ*B Pathway

**DOI:** 10.1155/2018/3692752

**Published:** 2018-08-19

**Authors:** Yazhou Lin, Guoqing Tang, Yucheng Jiao, Ye Yuan, Yuehuan Zheng, Yong Chen, Jiaqi Xiao, Changwei Li, Zhe Chen, Peng Cao

**Affiliations:** ^1^Department of Orthopedics, Ruijin Hospital, Shanghai Jiao Tong University School of Medicine, Shanghai 200025, China; ^2^Shanghai Key Laboratory for Prevention and Treatment of Bone and Joint Diseases with Integrated Chinese-Western Medicine, Shanghai Institute of Traumatology and Orthopedics, Ruijin Hospital, Shanghai Jiao Tong University School of Medicine, Shanghai 200025, China; ^3^Kunshan Hospital of Traditional Chinese Medicine, Kunshan 215300, China; ^4^Department of Orthopedics, Ruijin Hospital North, Shanghai Jiao Tong University School of Medicine, Shanghai 201800, China; ^5^Department of Medical Microbiology and Parasitology, Shanghai Jiao Tong University School of Medicine, Shanghai 200025, China

## Abstract

Accumulating evidence suggests that *Propionibacterium acnes* (*P. acnes*) is a novel pathogenic factor promoting intervertebral disc degeneration (IVDD). However, the underlying mechanisms by which *P. acnes* induces IVDD have been unclear. In this study, we quantified the severity of IVDD, as well as the expressions of inducible nitric oxide synthase (iNOS)/nitric oxide (NO) and cyclooxygenase (COX-2)/prostaglandin (PGE_2_) in human intervertebral discs (IVDs) infected with *P. acnes*. Compared with *P. acnes*-negative IVDs, *P. acnes*-positive IVDs showed increased iNOS/NO and COX-2/PGE_2_ activity concomitant with more severe IVDD. In order to detect the potential correlation between iNOS/NO expression, COX-2/PGE_2_ expression, and IVDD, we developed a *P. acnes*-induced IVDD rat model and found that the upregulation of iNOS/NO and COX-2/PGE_2_ was essential to the occurrence of *P. acnes*-induced IVDD. This finding was supported by the fact that the inhibition of iNOS/NO and COX-2/PGE_2_ activity ameliorated IVDD significantly, as evidenced by restored aggrecan and collagen II expression both *in vivo* and *in vitro*. Mechanistically, we found that *P. acnes* induced iNOS/NO and COX-2/PGE_2_ expressions via a reactive oxygen species- (ROS-) dependent NF-*κ*B cascade. Furthermore, NADPH oxidase participated in *P. acnes*-induced ROS, iNOS/NO, and COX-2/PGE_2_ expressions. Overall, these findings further validated the involvement of *P. acnes* in the pathology of IVDD and provided evidence that *P. acnes*-induced iNOS/NO and COX-2/PGE_2_ activation via the ROS-dependent NF-*κ*B pathway is likely responsible for the pathology of IVDD.

## 1. Introduction

Intervertebral disc degeneration (IVDD) is a prerequisite for a number of discogenic diseases and can produce many clinical symptoms including sciatica, low back pain, and physical dysfunction. All of these drastically affect patients' quality of life and productivity at work; they also significantly increase the burden of medical treatment [1]. Despite the common occurrence of IVDD, its pathogenesis is not fully understood.

Conventionally, excessive mechanical loading, nutritional disorder, traumatic injury, or genetic predisposition were considered the main etiologies for IVDD [1]. However, recent studies suggest that *Propionibacterium acnes* (*P. acnes*)—a low-virulence anaerobic bacterium found to reside latently within nonpyogenic intervertebral discs (IVDs)—can induce IVDD [2]. In patients with IVDD, the prevalence of *P. acnes* has ranged from 13% to 44% [3–7]. We also first identified the existence of the bacterium in IVDs via histologic observation [8]. Interestingly, our previous work suggested that patients with *P. acnes*-positive IVDs had more severe degeneration than those with *P. acnes*-negative IVDs [9, 10]. Furthermore, we found that the inoculation of *P. acnes* induced significant IVDD in animal models [11]. Therefore, *P. acnes* was considered to be one of the potential pathogenic factors of IVDD. However, until now, the mechanisms by which *P. acnes* causes IVDDs have not been fully understood. Our previous study revealed that *P. acnes* can induce IVDD by promoting apoptosis of the nucleus pulposus [10]. But other possible pathologic mechanisms remained to be explored.

Until now, the mechanisms by which *P. acnes* causes IVDDs have not been fully understood. Although our previous study revealed that *P. acnes* can induce IVDD by promoting apoptosis of the nucleus pulposus, inhibition of *P. acnes*-induced apoptosis of nucleus pulposus cells (NPCs) only partly relieves the IVDD [10], suggesting that there may be other pathophysiological mechanisms of *P. acnes*-induced IVDD. Therefore, whether *P. acnes* would cause the IVDD via other possible mechanism is an interesting problem worth investigating.

Nitric oxide (NO) and prostaglandin E_2_ (PGE_2_) are two well-known catabolic factors involved in the occurrence and development of IVDD [12, 13]. When stimulated, the activation of iNOS and COX-2 results in the production of NO and PGE_2_ in IVDs and causes harmful pathophysiologic effects such as inflammation, apoptosis, and degeneration [13–16]. Interestingly, *P. acnes* is able to stimulate various cells, such as macrophages and keratinocytes, to produce NO and PGE_2_ [17]. An important question, therefore, was whether *P. acnes* could promote the expression of NO and PGE_2_ in IVDs and thus induce IVDD.

We first sought to determine whether latent infection with *P. acnes* was responsible for IVDD by promoting the production of NO and PGE_2_. In addition, the underlying signaling pathway involving this process was further explored. To our knowledge, this is the first study to investigate the relationship between *P. acnes* infection and NO/PGE_2_, and our findings provide new insights for the prevention and treatment of degenerative disc diseases.

## 2. Materials and Methods

### 2.1. Patients, Bacterial Culture, and Match of Samples

A total of 46 patients were included in this study from September 2013 to May 2017. The patients underwent discectomy at the single-level lumbar spine due to intervertebral disc degeneration associated with low back pain and/or sciatica. All patients were assigned for surgery after failed attempts to improve their condition using conservative treatment for several months. Patients who received antibiotics within the month preceding surgery were not included. According to our previous protocol [8], all tissues were cultured in tryptone soy broth (TSB) for 14 days under anaerobic conditions, then the presence of bacteria in the culture was identified by amplifying the 16S rDNA gene by PCR.

According to previous study, the variables age, primary symptoms, duration of symptoms, and surgery level dramatically affected the severity of IVDD. Thus, to reveal the exact pathological effect of *P. acnes* and reduce heterogeneity, a case-controlled method was used for the quantitative analysis following a previous study [18]. After culture of the specimen and identification of the bacteria, the patients who had *P. acnes* only in IVDs were classified as the positive group (*n* = 23). Equal numbers of patients who were identified as completely bacteria-free in IVDs were selected to match each of the positive patients based on the following criteria: (1) same gender, (2) same surgery segment, (3) same symptoms of low back pain only, sciatica only, or both, (4) similar ages ± 5 years, and (5) similar duration of symptoms ± 3 months. These patients were named the negative group (*n* = 23).

### 2.2. Preparation of *P. acnes* Inoculum

A standard strain of *P. acnes* (ATCC: 6919, GIM: 1.243, Guangdong Microbiology Culture Center, Guangdong, China) was cultured on Gifu Anaerobic (GAM) broth (Nissui, Tokyo, Japan) for 3 d at 37°C under anaerobic conditions.

### 2.3. Inoculation of *P. acnes* into Caudal IVDs of Rat

According to the previous study [10], the target vertebrae (Ca)6/7 to (Ca)8/9 (*n* = 3 per animal) of eight-week-old male Sprague-Dawley rats were identified and marked by palpation and X-ray before surgery. A volume of 2.5 *μ*l *P. acnes* (OD_600_ = 3.0), *P. acnes* with N^G^-monomethyl-L-arginine, monoacetate salt (L-NMMA, iNOS inhibitor, 1 mM, no. S0011, Beyotime, Shanghai, China), *P. acnes* with diclofenac sodium (DS, COX-2 inhibitor, 200 *μ*M, Selleck, Houston, USA), or saline was inoculated vertically into the nucleus pulposus using a microsyringe with a 28-gauge needle (Hamilton, Nevada, USA). All animal experiments were performed in accordance with the protocol approved by the Shanghai Jiao Tong University (SJTU) Animal Care and Use Committee [IACUC protocol number: SYXK (Shanghai)2011-0113] and in accordance with the Ministry of Science and Technology of the People's Republic of China Animal Care guidelines. All surgeries were performed under anesthesia, and all efforts were made to minimize suffering.

### 2.4. Cocultures of NPCs and *P. acnes*

Tissues of nucleus pulposus were harvested and cultured from 5 disc degenerated patients, including 3 males and 2 females, with a mean age of 36.5 years (28–50 years) following the above protocol. Cell samples from different patients were kept separate. All experiments were carried out in duplicate and were conducted with human NPCs from passages 2 to 3.

For coculture, the bacteria were harvested from the third day cultures in stationary phase and washed twice with phosphate-buffered saline (PBS). The bacterial density was adjusted to optical density (OD_600_ = 2). Then, *P. acnes* were added to the cell culture (5 × 10^5^ cells/well) in a 6-well culture plate at a 100 : 1 multiplicity of infection (MOI) without antibiotics. After 12 and 24 hours, cocultured cells were washed three times with PBS and prepared for late-stage experiments.

### 2.5. Western Blot Analysis

For Western blot analysis, total proteins from the samples were separated by SDS-PAGE, transferred to nylon membranes, and incubated separately with the following primary antibodies: collagen II (dilution of 1 : 2000; cat. number ab34712, Abcam, Britain), aggrecan (dilution of 1 : 1000; cat. number ab36861, Abcam, Britain), iNOS (dilution of 1 : 500; cat. number ab3523, Abcam, Britain), COX-2 (dilution of 1 : 1000; cat. number 12282, CST Inc., MA, USA), and NF-*κ*Β/phospho-NF-*κ*Β (dilution of 1 : 1000; cat. number 8242S/3033S, CST Inc., MA, USA). *β*-Actin (dilution of 1 : 2000; cat. number CW0096, CW Bio, Beijing, China) was used as an internal control. Then, the membranes were incubated with horseradish peroxidase-conjugated secondary antibody, goat anti-rabbit IgG (dilution of 1 : 2000; cat. number CW0103s, CW Bio, Beijing, China), or goat anti-mouse IgG (dilution of 1 : 2000; cat. number CW0102s, CW Bio, Beijing, China) at room temperature for 2 h, and the bands were visualized using chemiluminescence (Pierce Biotechnology Inc., IL, USA). The images were analyzed using Fusion FX7 (Vilber Lourmat, Marne-la-Vallée, France).

### 2.6. Immunofluorescence

ROS level was quantified using dihydroethidium (DHE, 5 *μ*M, S0063, Beyotime, Shanghai, China). NPCs were treated with DHE and incubated for 30 min at 37°C.

For iNOS and COX-2, *P. acnes*-induced cells were cultured on glass slides and then fixed for 30 min in 4% paraformaldehyde. The NPCs were incubated for 16 h at 4°C with iNOS (dilution of 1 : 500, cat. number Ab178945, Abcam, Britain) and COX-2 antibody (dilution of 1 : 1000, cat. number 12282, CST Inc., MA, USA). All images were observed using a fluorescence microscope (Axio, Carl Zeiss, Oberkochen, Germany).

### 2.7. Flow Cytometric Analysis

ROS level was determined using dichloro-dihydro-fluorescein diacetate (DCFH-DA, 10 *μ*M, S0033, Beyotime, Shanghai, China) and directly treated with 2 *μ*l of DCFH-DA (10 mM) dissolved in PBS (2 ml) at 37°C for 20 min. Fluorescence was analyzed using a FACScan (Becton Dickinson, Sunnyvale, CA) flow cytometer with excitation at 488 nm and emission at 530 nm.

The proportion of NPC apoptosis was detected using the Annexin V-FITC apoptosis detection kit (C1063, Beyotime, Shanghai, China) and calculated by the percentage of early apoptotic (Annexin V+/PI−) cells plus the percentage of late apoptotic (Annexin V+/PI+) cells using flow cytometry.

### 2.8. Measurement of PGE2 Production

Nucleus pulposus cells (NPCs) were subcultured in 6-well plates, cocultured with *P. acnes* for different times and incubated with indicated components, respectively. The supernatant (1 ml) of the culture medium was collected to determine PGE_2_ concentrations by an enzyme-linked immunosorbent assay (ELISA) (SKGE004B, R&D Systems Inc., Minneapolis, MN, USA).

### 2.9. Nitrite Assay

The nitrite concentration in the medium was measured as an indicator of NO production according to the Griess reaction [19]. A volume of 50 *μ*l of each supernatant was mixed with the same volume of Griess reagent (S0021, Beyotime, Shanghai, China), and the absorbance of the mixture at 550 nm was determined with an ELISA plate reader (Dynatech MR-7000; Dynatech Laboratories).

### 2.10. Statistical Analysis

Data were collected from three or more independent experiments and expressed as the mean ± SD. A two-sided Student's *t*-test was used to analyze differences between two groups. One-way ANOVA was performed to show differences among multiple groups. *P* < 0.05 was considered significantly different.

## 3. Results

### 3.1. IVDs Infected with *P. acnes* Had Increased iNOS/NO and COX-2/PGE_2_ Expressions Concomitant with Severe Disc Degeneration in Patients

**Figure 1:**
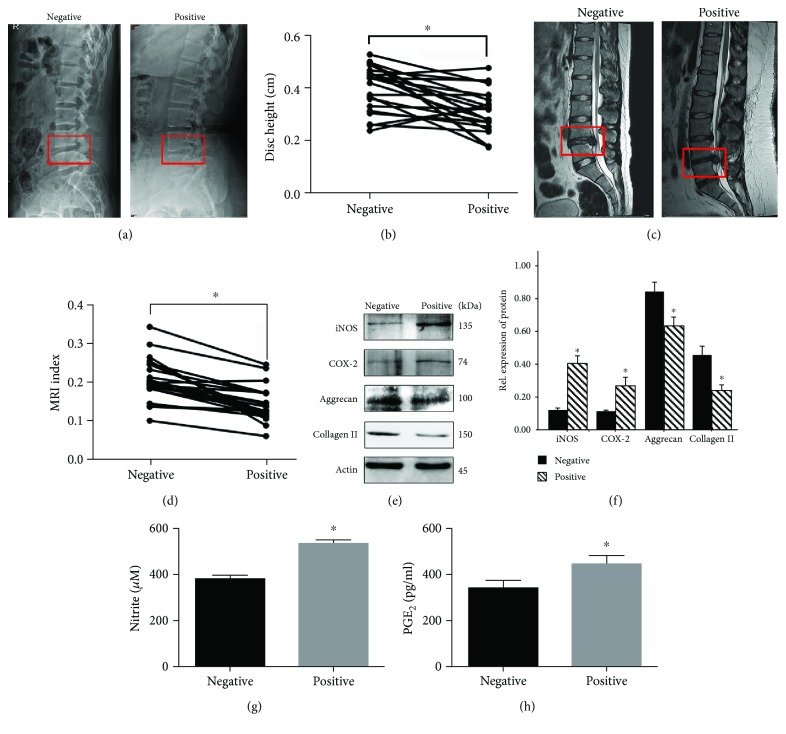
The IVDs infected with *P. acnes* had increased iNOS/NO and COX-2/PGE_2_ expressions, concomitant with severe disc degeneration. (a–d) Representative images from lateral X-rays and magnetic resonance imaging point to severe degeneration in *P. acnes*-positive IVDs (*n* = 23 for each group). (e–f) Western blot analysis of aggrecan, collagen II, iNOS, and COX-2 in human IVDs. ^∗^The *P. acnes*-positive groups compared with the *P. acnes*-negative groups. (g, h) The concentration of NO and PGE_2_ were tested by the Griess reaction and ELISA, respectively. ^∗^The *P. acnes*-positive groups compared with the *P. acnes*-negative groups; *P* < 0.05. *P* values were analyzed by paired *t*-test, Student's *t*-test, and one-way ANOVA. Data are presented as the mean ± SD from three independent experiments.

To explore the role of iNOS and COX-2 in *P. acnes*-induced IVDD, we first evaluated iNOS and COX-2 expressions in human IVDs infected with *P. acnes*. The radiographic analysis suggested that intervertebral height in the *P. acnes*-positive group was much lower than that in the *P. acnes*-negative group (Figures 1(a) and 1(b)). Moreover, magnetic resonance imaging (MRI) showed more hypointense signals in the midsagittal T2-weighted images of *P. acnes*-positive IVDs (Figures 1(c) and 1(d)) than in images of *P. acnes*-negative IVDs, thus further demonstrating that *P. acnes* infection is associated with more severe IVDD.

In addition, quantitative analysis of protein suggested that the expression of iNOS and COX-2 increased significantly in the *P. acnes*-positive group compared with the *P. acnes*-negative group (Figures 1(e) and 1(f)). This was accompanied by a decrease in the expression of aggrecan and collagen II (Figures 1(e) and 1(f)) and an increase in the concentration of NO and PGE_2_ (Figures 1(g) and 1(h)). Taken together, these results show that *P. acnes* infection increases iNOS/NO and COX-2/PGE_2_ expressions in human IVDs, leading to the conclusion that *P. acnes* infection can promote the degeneration of IVDs by increasing iNOS/NO and COX-2/PGE_2_ activity.

### 3.2. *P. acnes* Infection Induced IVDD by Promoting the Expression of iNOS/NO and COX-2/PGE_2_

**Figure 2:**
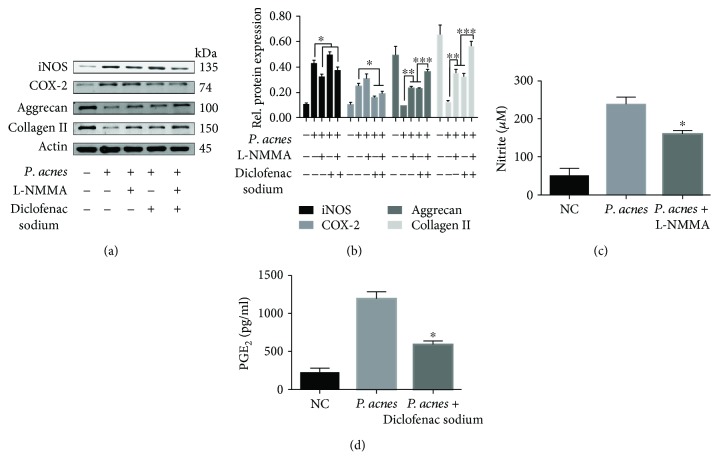
Caudal IVD inoculation with *P. acnes*-induced IVDD by promoting iNOS/NO and COX-2/PGE_2_ expression in rats. (a, b) Western blot analysis of aggrecan, collagen II, iNOS, and COX-2 expression in the IVDs of rats inoculated with *P. acnes* for 72 h, with or without L-NMMA (1 mM) and DS (200 *μ*M) pretreatment. ^∗^/^∗∗^The infected group compared with the infection + L-NMMA and/or DS groups. ^∗∗∗^The infection + L-NMMA and DS group compared with the infection + L-NMMA or DS groups. (c, d) The concentration of NO and PGE_2_ as tested in different groups. ^∗^The infected group compared with the infection + L-NMMA or DS groups; *P* < 0.05. *P* values were analyzed by Student's *t*-test and one-way ANOVA. Data are presented as the mean ± SD from three independent experiments.

To further elucidate the relationship between iNOS/NO, COX-2/PGE_2_ activity, and IVDD caused by *P. acnes* infection, the bacteria were inoculated into the caudal IVDs of rats. After 72 hours, quantitative protein analysis showed that the expression of iNOS/NO and COX-2/PGE_2_ increased significantly in *P. acnes*-inoculated IVDs compared with control samples (Figures 2(a)–2(d)), whereas the expression of aggrecan and collagen II decreased significantly (Figures 2(a) and 2(b)). More importantly, when iNOS/NO and COX-2/PGE_2_ activation was inhibited by L-NMMA (inhibitor of iNOS) and diclofenac sodium (inhibitor of COX-2), respectively (Figures 2(a)–2(d)), *P. acnes*-induced IVDD was ameliorated, as evidenced by the partially restored expression of aggrecan and collagen II (Figures 2(a) and 2(b)). Interestingly, we found that iNOS and COX-2 exerted a synergistic effect in the promotion of IVDD, as the simultaneous application of L-NMMA and diclofenac sodium restored the expression of aggrecan and collagen II to a higher level than when L-NMMA or diclofenac sodium was used alone (Figures 2(a) and 2(b)). Collectively, these results show that iNOS/NO and COX-2/PGE_2_ activation is essential for *P. acnes*-decreased aggrecan and collagen II expression during IVDD *in vivo*.

**Figure 3:**
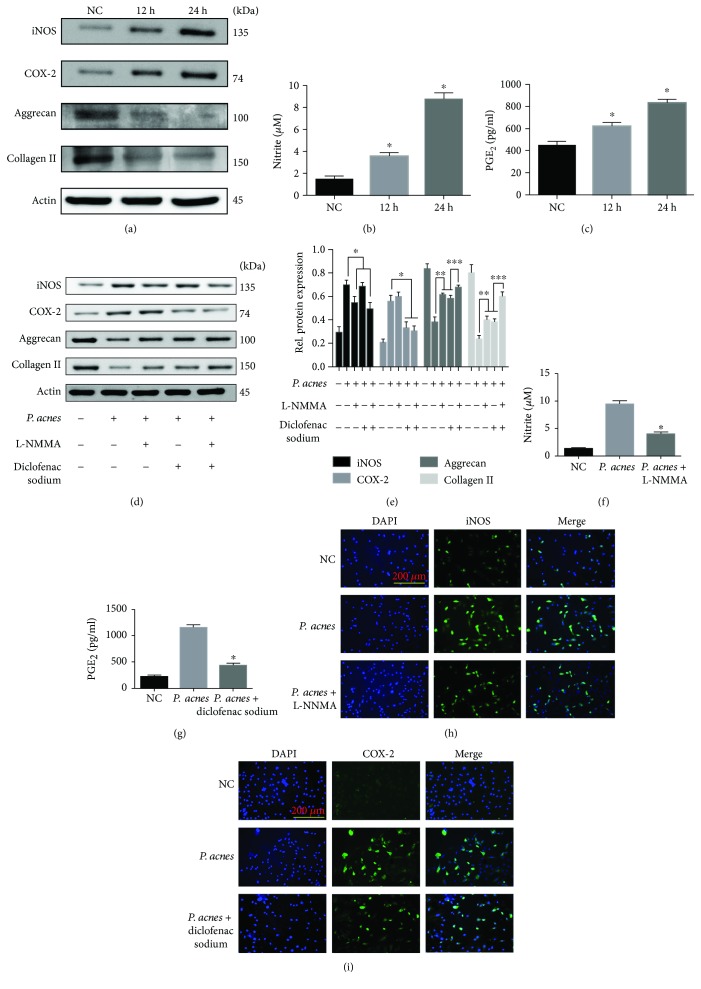
Inoculation with *P. acnes* induced iNOS/NO and COX-2/PGE_2_ expressions in NPCs. (a) Time curve expression of iNOS, COX-2, aggrecan, and collagen II in NPCs induced by *P. acnes* at a MOI = 100 : 1. (b, c) *P. acnes* inoculation increased NO and PGE_2_ production in a time-dependent manner. ^∗^The groups with different incubation times compared with the negative control group. (d, e) Western blot analysis of iNOS, COX-2, aggrecan, and collagen II expression in NPCs infected with *P. acnes* for 24 hours, pretreated with or without L-NMMA (100 *μ*M) and DS (200 nM). ^∗^/^∗∗^The infected group compared with the infection + L-NMMA and/or DS groups. ^∗∗∗^The infection + L-NMMA and DS group compared with the infection + L-NMMA or DS groups. (f, g) The concentration of NO and PGE_2_ production induced by *P. acnes* infection for 24 hours, pretreated with or without L-NMMA (100 *μ*M) and DS (200 nM). ^∗^The infection group compared with the infection + L-NMMA or DS groups. (h, i) Immunofluorescence analysis of iNOS and COX-2 expressions in NPCs induced by *P. acnes* infection for 24 hours, pretreated with or without L-NMMA (100 *μ*M) and DS (200 nM); *P* < 0.05. *P* values were analyzed by one-way ANOVA. Data are presented as the mean ± SD from three independent experiments.

Next, we sought to investigate whether *P. acnes* could inhibit aggrecan and collagen II expression by promoting iNOS/NO and COX-2/PGE_2_ expressions *in vitro*. The results showed that *P. acnes* incubation (MOI = 100) increased iNOS/NO and COX-2/PGE_2_ expressions (Figures 3(a)–3(c)), whereas it decreased aggrecan and collagen II expressions in a time-dependent manner in nucleus pulposus cells (NPCs) (Figure 3(a)). However, these processes were significantly dampened in the presence of L-NMMA (100 *μ*M) and diclofenac sodium (200 nM) (Figures 3(d)–3(i)). Taken together, these results show that *P. acnes* can induce the degeneration of NPCs by promoting iNOS/NO and COX-2/PGE_2_ activity *in vitro*.

### 3.3. *P. acnes* Infection Induced iNOS/NO and COX-2/PGE_2_ Production via the Generation of ROS

**Figure 4:**
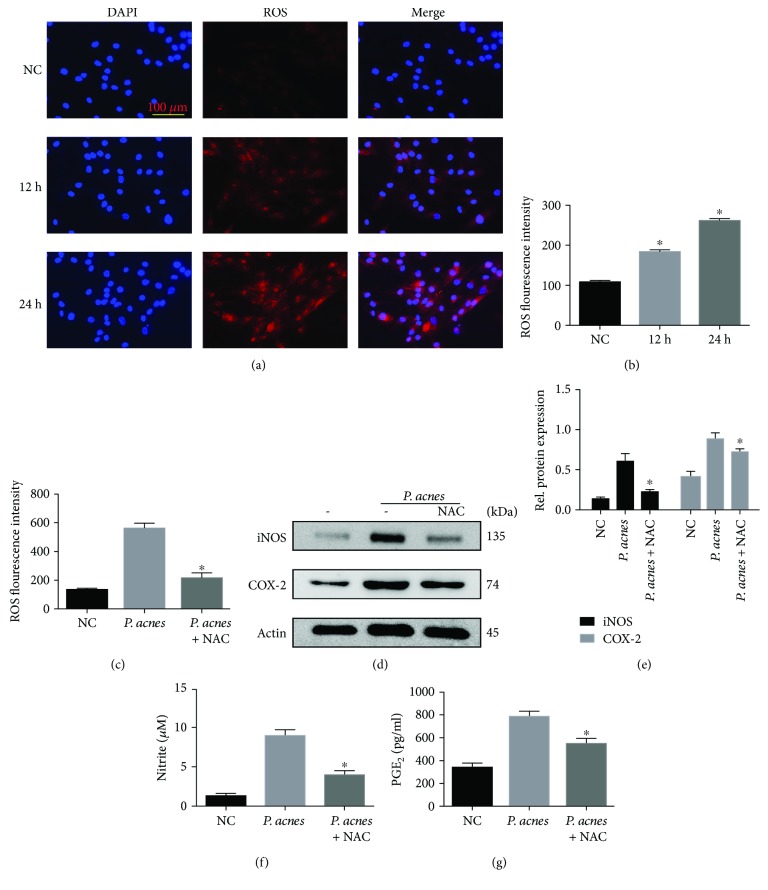
*P. acnes* induced iNOS/NO and COX-2/PGE_2_ production via increased ROS production in NPCs. (a) Immunofluorescence analysis of ROS in NPCs in response to *P. acnes* infection at a MOI = 100 : 1 for different time periods. (b) The ROS fluorescence intensity was tested by flow cytometric analysis. ^∗^The different incubation time groups compared with the negative control group. (c–g) The flow cytometric analysis of ROS, Western blot of iNOS and COX-2, and the production of NO and PGE_2_ in NPCs infected with *P. acnes* for 24 hours, with or without NAC (10 mM) preincubation. ^∗^The infection + NAC group compared with the infection group. *P* < 0.05; *P* values were analyzed by one-way ANOVA. Data are presented as the mean ± SD from three independent experiments.

Having established the critical role of iNOS and PGE_2_ in *P. acnes*-induced IVDD, we next sought to explore the molecular mechanisms involved in the induction of iNOS and PGE_2_ by *P. acnes* in NPCs. Since ROS have been shown to regulate iNOS and COX-2 expressions in various cells [20], we hypothesized that ROS generation was required for iNOS and PGE_2_ expressions induced by *P. acnes*. To test this, we first detected the intracellular ROS levels in NPCs infected with *P. acnes* by DHE probe and DCFH-DA staining for 12 and 24 hours. The results showed that the incubation of *P. acnes* increased the intracellular ROS levels in a time-dependent manner (Figures 4(a) and 4(b)). To detect the causal role of ROS in iNOS and COX-2 expressions, the ROS scavenger N-acetyl-L-cysteine (NAC) was used. The results showed that pretreatment with NAC significantly reduced ROS levels induced by *P. acnes* (Figure 4(c)). Concomitantly, iNOS and COX-2 expressions, as well as NO and PGE_2_ production, were all abundantly dampened after NAC application (Figures 4(d)–4(g)). Taken together, these results indicate that an increase in ROS is essential for *P. acnes*-induced iNOS/NO and COX-2/PGE_2_ expressions in NPCs.

### 3.4. *P. acnes* Induced iNOS/NO and COX-2/PGE_2_ Expressions via the ROS-Dependent NF-*κ*B Cascade

**Figure 5:**
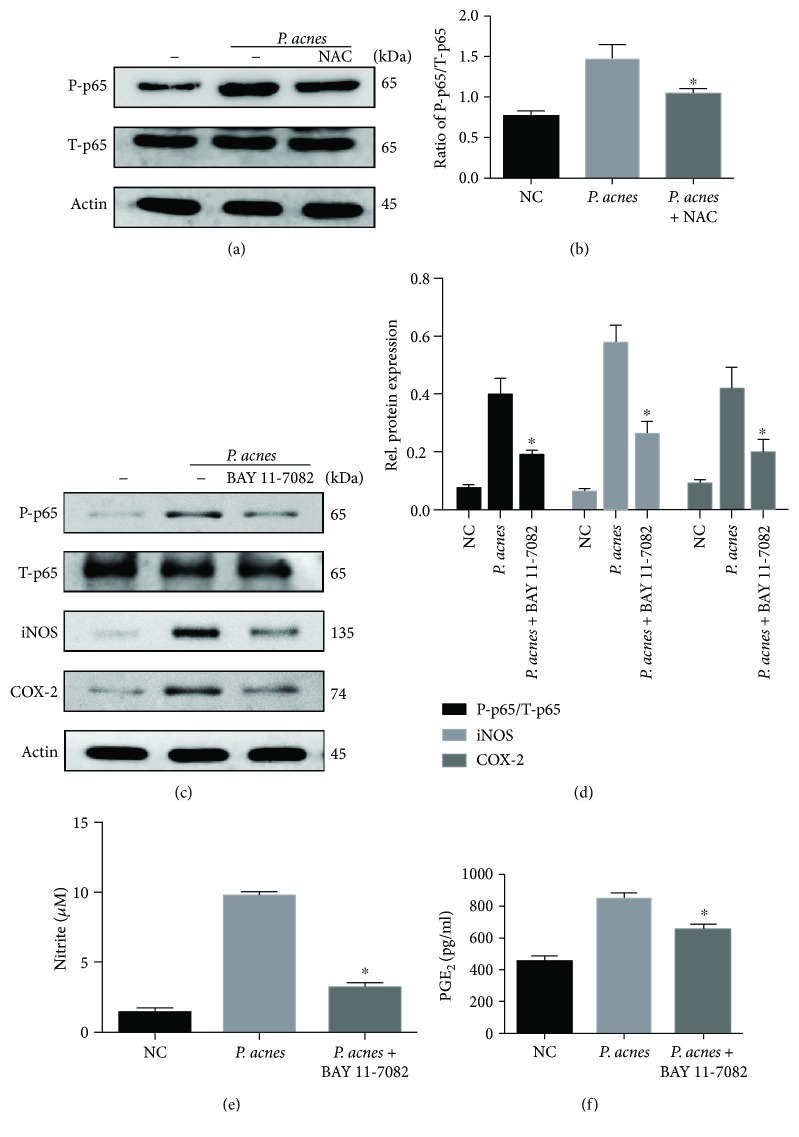
*P. acnes* induced iNOS/NO and COX-2/PGE_2_ expressions via a ROS-dependent NF-*κ*B cascade. (a, b) Western blot analysis of NF-*κ*B p65 activation in the presence of *P. acnes* for 1 hour, pretreated with or without NAC (10 mM) in NPCs. (c–f) The activation of NF-*κ*B p65, expression of iNOS and COX-2 (c, d), and production of NO and PGE_2_ (e, f) were analyzed. The NPCs were incubated with *P. acnes* for 1 hour (for NF-*κ*B p65 activation) and 24 hours (for iNOS, COX-2, NO, and PGE_2_ expression), pretreated with or without NF-*κ*B-specific inhibitors (BAY 11-7082, 10 *μ*M) for 1 hour. ^∗^The infection + BAY 11-7082/NCA group compared with the infection group; *P* < 0.05. *P* values were analyzed by one-way ANOVA. Data are presented as the mean ± SD from three independent experiments. P-p65: phosphorylated-p65; T-p65: total-p65.

We further explored the ROS-mediated downstream signaling pathways of iNOS/NO and COX-2/PGE_2_ expressions. Since our previous report demonstrated that the NF-*κ*B signaling pathway mediated *P. acnes*-induced IVDD by regulating IL-1*β* and TNF-*α* expressions [10], we speculated that NF-*κ*B activation might be essential for ROS-induced iNOS/NO and COX-2/PGE_2_ expressions. The results show that ROS scavenger NAC significantly inhibited NF-*κ*B activation induced by *P. acnes* (Figures 5(a) and 5(b)), demonstrating that the NF-*κ*B pathway was downstream of ROS in NPCs.

Next, we sought to determine whether NF-*κ*B activation was critical for ROS-induced iNOS/NO and COX-2/PGE_2_. The results show that in line with the dampened p65 phosphorylation, iNOS and COX-2 expressions (Figures 5(c) and 5(d)) as well as the concentration of NO and PGE_2_ were significantly decreased by NF-*κ*B inhibitor-BAY11 pretreatment (Figures 5(e) and 5(f)). Taken together, the data demonstrate that ROS-dependent NF-*κ*B activation contributes to *P. acnes*-induced iNOS/NO and COX-2/PGE_2_ activation in NPCs.

### 3.5. NADPH Oxidase Participates in *P. acnes*-Induced ROS, iNOS/NO, and OX-2/PGE_2_ Expressions

**Figure 6:**
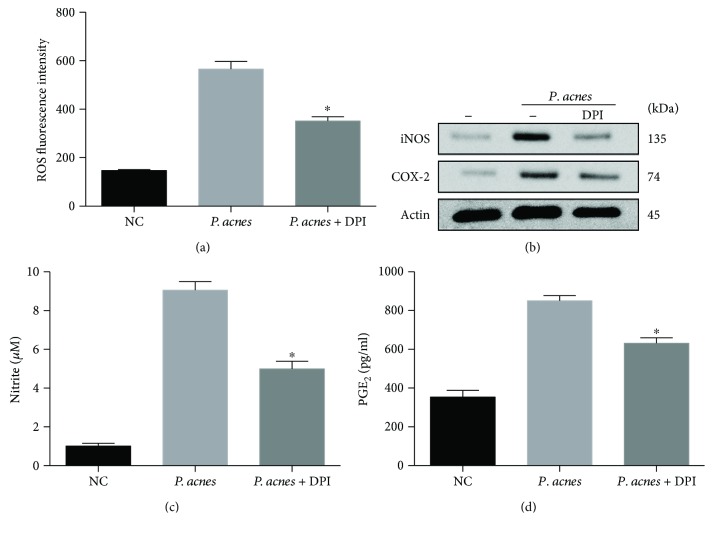
Participation of NADPH oxidase in *P. acnes*-induced ROS, iNOS/NO, and COX-2/PGE_2_ expressions. (a) Flow cytometric analysis of ROS fluorescence intensity in NPCs infected with *P. acnes* for 24 hours, pretreated with or without DPI (10 *μ*M). ^∗^The infection + DPI group compared with the infection group. (b–d) DPI inhibited *P. acnes*-induced iNOS/NO and COX-2/PGE_2_ production in NPCs. ^∗^The infection + DPI group compared with the infection group; *P* < 0.05. *P* values were analyzed by one-way ANOVA. Data are presented as the mean ± SD from three independent experiments.

Since NADPH oxidase is an enzymatic source for the production of ROS under various pathologic conditions [21], we next wanted to establish the role of NADPH oxidase in *P. acnes*-induced ROS, iNOS, and COX-2 expressions. The quantitative results of DCFH-DA-positive cells via flow cytometric analysis showed that treatment with the NADPH oxidase inhibitor diphenyliodonium chloride (DPI) significantly decreased the ROS levels of NPCs induced by *P. acnes* (Figure 6(a)). In line with the change of intracellular ROS levels, iNOS and COX-2 expressions, as well as the increased concentrations of NO and PGE_2_ induced by *P. acnes*, were significantly dampened by the application of DPI (inhibitor of NADPH oxidase) (Figures 6(b)–6(d)). Taken together, these data indicate that activation of NADPH oxidase contributed to *P. acnes*-induced ROS production as well as iNOS/NO and COX-2/PGE_2_ expressions in NPCs.

## 4. Discussion

Latent infection with *P. acnes* has been thought to be crucial to the pathogenesis of IVDD, and the relationship between *P. acnes* and IVDD has gradually become a hot spot for research [8–11]. More importantly, we found that the pathologic mechanism of *P. acnes*-induced IVDD was the activation of iNOS/NO and the COX-2/PGE_2_ system in NPCs. Finally, the signaling pathway involved in the activation of the iNOS/NO and COX-2/PGE_2_ system by *P. acnes* has proved to be the ROS-dependent NF-*κ*B pathway.

iNOS and COX-2 are genes that produce NO and PGE_2_ in cells; they are not only cellular signaling factors but also well-known pathogenic factors for IVDD. Both NO and PGE_2_ are able to inhibit the synthesis of aggrecan in NPCs, which then leads to the destruction of extracellular matrix in IVDs [15, 22]. In addition, increased PGE_2_ affects the catabolic and anabolic balance in IVDs—for example, by promoting the expression of matrix metalloproteinase (MMP) and inhibiting the expression of insulin-like growth factor-1 (IGF-1) as well as accelerating the degeneration of IVDs [16]. Also, NO is a proapoptotic factor for NPCs, and the apoptosis of NPCs has been proved to be the key cause of IVDD [14]. However, whether NO and PGE_2_ are involved in *P. acnes*-induced IVDD is unknown. We found that *P. acnes* induced IVDD as well as the overexpression of iNOS/NO and COX-2/PGE_2_ *in vivo* and *in vitro*, whereas elimination of iNOS and COX-2 activity significantly decreased NO and PGE_2_ production while also ameliorating IVDD. More interestingly, inhibition of both NO and PGE_2_ generation simultaneously was more effective than inhibiting either one alone, suggesting that the two factors have a synergetic effect in promoting *P. acnes*-induced IVDD. Therefore, it is reasonable to conclude that *P. acnes-*induced iNOS/NO and *P. acnes-*induced COX-2/PGE_2_ are direct factors leading to the development of IVDD. Since our precious report has demonstrated that *P. acnes* infection increases NPC degeneration via inducing the apoptosis of NPCs [10], we next detected whether the inhibition of iNOS and COX-2 activity had an effect on the survival of NPCs. The results (Supplemental Figure 1) showed that L-NMMA and diclofenac sodium treatment significantly decreased *P. acnes*-induced apoptosis of NPCs. In addition, we found that L-NMMA and diclofenac sodium exerted a synergistic effect on the inhibition of NPC apoptosis induced by *P. acnes*, as the simultaneous application of L-NMMA and diclofenac sodium reduced the apoptosis percentage to a lower level than that in the groups where L-NMMA or diclofenac sodium was used alone. Taken together, these results demonstrated that iNOS- and COX-2-mediated NPC degeneration might also decrease the apoptosis of NPCs induced by *P. acnes*.

With further investigation, we found that the activation of *P. acnes-*induced iNOS/NO and COX-2/PGE_2_ was mediated by ROS. ROS are a family of unstable and highly reactive molecules with or without free radicals, mainly including superoxide anion (O_2_^−^), hydrogen peroxide (H_2_O_2_). ROS are inevitably produced via the oxygen-using metabolic process [23]. Previous studies have demonstrated that keratinocytes and macrophages generate many ROS with *P. acnes* stimulation [17]. When we cocultured NPCs with *P. acnes*, the concentration of ROS significantly increased, along with an increase of iNOS/NO and COX-2/PGE_2_, whereas excessive iNOS/NO and COX-2/PGE_2_ expressions were significantly decreased when ROS were neutralized by NAC (Figures 4(d)–4(f)), suggesting that ROS are the key molecules involved in *P. acnes*-facilitated iNOS/NO and COX-2/PGE_2_ activation.

There are two main sources of ROS in cells: the mitochondrial and nonmitochondrial pathways [21, 24]. In the nonmitochondrial pathway, the ROS are mainly generated from the cytosolic enzymes of NADPH oxidase. In this study, the concentration of ROS significantly decreased when the activity of NADPH oxidase was inhibited (Figure 6(a)), suggesting that the *P. acnes*-induced ROS stem primarily from the nonmitochondrial pathway. However, our previous study proved that *P. acnes* could activate the TLR-2/JNK/mitochondrial pathway during the apoptosis of NPCs [10], and dysfunction of the mitochondrion was shown to be another source of ROS in many cells [25, 26]. Therefore, we speculated that some ROS may arise from the mitochondrial dysfunction induced by *P. acnes*, which corresponds with the finding that the NADPH oxidase inhibitor DPI cannot completely scavenge the ROS production induced by *P. acnes*.

Accumulated ROS appear to regulate cellular function via downstream signaling pathways, such as NF-*κ*B [27, 28]. NF-*κ*B is an important transcriptional factor that plays pivotal roles in inflammatory responses by regulating genes that encode proinflammatory proteins such as iNOS and COX-2 [29]. However, whether activation of the NF-*κ*B pathway is critical for iNOS and COX-2 activation induced by ROS in the presence of *P. acnes* is less clear. We found that accumulated ROS increased the phosphorylation of NF-*κ*B p65 and inhibited NF-*κ*B p65 with BAY 11-7082 pretreatment, thus alleviating the overexpression of iNOS/NO and COX-2/PGE_2_. These findings suggest that ROS mediate iNOS/NO and COX-2/PGE_2_ via NF-*κ*B p65 in *P. acnes*-stimulated NPCs.

**Figure 7:**
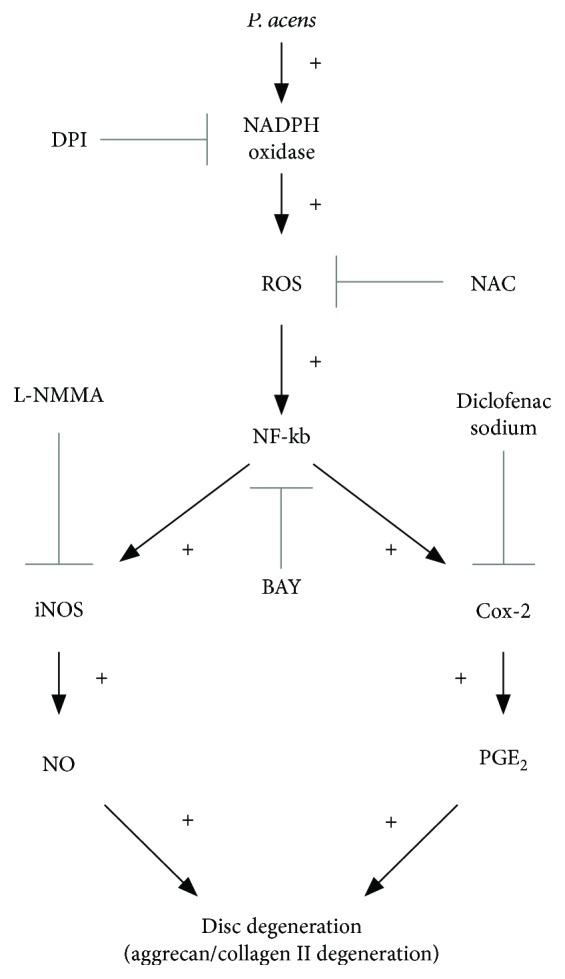
Schematic illustration of *P. acnes*-induced IVDD by promoting iNOS/NO and COX-2/PGE2.

In conclusion, this study demonstrates that *P. acnes* induces IVDD by promoting iNOS/NO and COX-2/PGE_2_ activation via a ROS-dependent NF-*κ*B pathway (Figure 7). The confirmation of *P. acnes* as a pathogenic factor for IVDD and elucidation of the underlying mechanisms provide new insights into IVDD and may ultimately lead to the development of new treatment regimens for IVDD.

## Data Availability

The data used to support the findings of this study are available from the corresponding author upon request.

## Abbreviations

*P. acnes*: *Propionibacterium acnes*
IVD: Intervertebral disc
IVDD: Intervertebral disc degeneration
NPCs: Nucleus pulposus cells
MOI: Multiplicity of infection
DS: Diclofenac sodium
DCFH-DA: Dichloro-dihydro-fluorescein diacetate
L-NMMA: N^G^-monomethyl-L-arginine, monoacetate salt
DPI: Diphenyliodonium chloride
MMP: Matrix metalloproteinase
IGF-1: Insulin-like growth factor-1.

## Supplementary Materials

Supplementary Materials Supplemental Figure 1: the inhibition of iNOS and/or COX-2 prevents apoptotic cell death of NPCs. Flow cytometric analysis of apoptosis in NPCs infected with *P. acnes* for 24 hours, pretreated with or without L-NMMA (100 *μ*M) and DS (200 nM). DS: diclofenac sodium.
