# Supplementary material for: Propionibacterium acnes Induces Intervertebral Disc Degeneration by Promoting iNOS/NO and COX-2/PGE2 Activation via the ROS-Dependent NF-κB Pathway
Source: Oxid Med Cell Longev. 2018 Aug 19;2018:3692752. doi: 10.1155/2018/3692752 (PMC6120277; doi:10.1155/2018/3692752)
Supplement: Supplementary Materials — Supplemental Figure 1: the inhibition of iNOS and/or COX-2 prevents apoptotic cell death of NPCs. Flow cytometric analysis of apoptosis in NPCs infected with P. acnes for 24 hours, pretreated with or without L-NMMA (100 μM) and DS (200 nM). DS: diclofenac sodium. [file 3692752.f1.pdf]

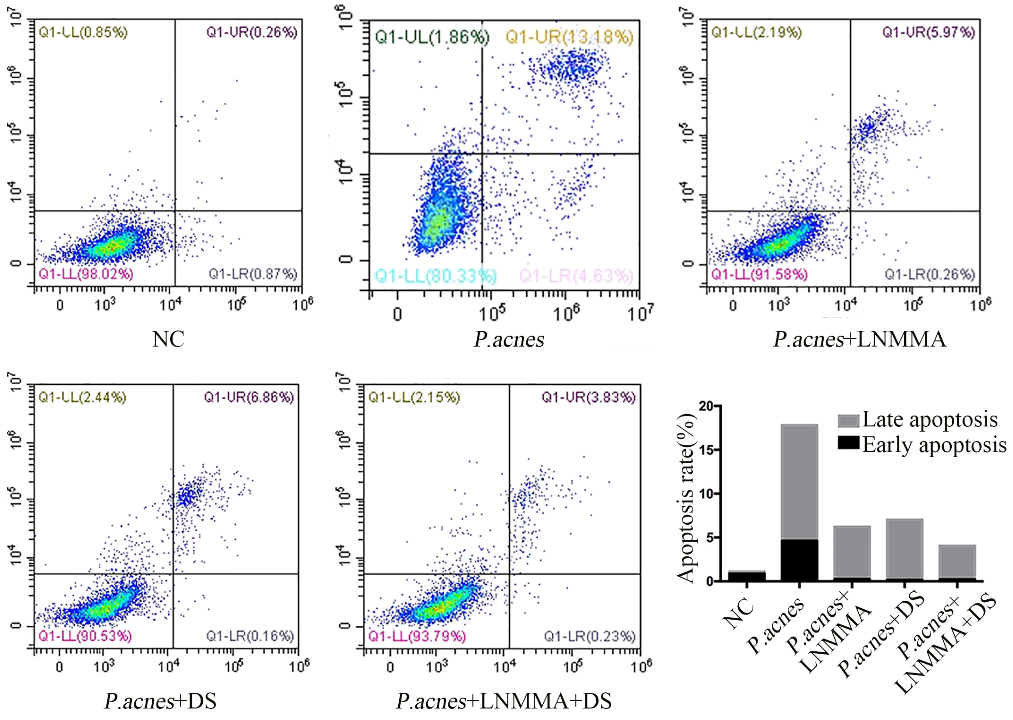

Supplemental 1. The inhibition of iNOS and/or COX-2 prevent apoptotic cell death of NPCs. Flow cytometric analysis of apoptosis in NPCs infected with *P. acnes* for 24 hours, pretreated with or without L-NMMA (100 μM) and DS (200 nM). DS: Diclofenac Sodium
